# Sleep Quality and Nicotine Dependence Among Lebanese University Residents and Fellows: A Cross-Sectional Study

**DOI:** 10.7759/cureus.42364

**Published:** 2023-07-24

**Authors:** Elias M Nabhan, Kamel Jaafar, Rama Daoud, Zeina Nasser

**Affiliations:** 1 Internal Medicine, Lebanese University Faculty of Medicine, Beirut, LBN; 2 Cardiology, University of Balamand, Beirut, LBN; 3 Obstetrics and Gynaecology, Lebanese University Faculty of Medicine, Beirut, LBN; 4 General Medicine, Lebanese University Faculty of Medicine, Beirut, LBN; 5 Faculty of Medical Sciences, Neuroscience Research Center (NRC), Lebanese University, Hadath, LBN

**Keywords:** lebanon, cigarettes, waterpipe, iqos, nicotine dependence, sleep quality, pittsburg sleep quality index (psqi)

## Abstract

Background and objectives

Sleep quality and nicotine addiction are important public health issues with significant negative impacts on individual well-being and the performance of healthcare professionals. This study aims to determine the prevalence and association of nicotine dependence and poor sleep quality among residents and fellows enrolled in the Lebanese University.

Methods

A cross-sectional study using the snowball-sampling technique was conducted in Lebanon between January and March 2023. Data were collected through an online survey that included information on socio-demographic characteristics, nicotine dependence, and sleep quality. A total of 350 residents and fellows were included in the study. Bivariate analysis and multivariable logistic regression were carried out to identify the factors associated with sleep quality. Adjusted odds ratio and 95% confidence intervals were reported.

Results

One quarter (25.1%) of the residents and fellows were smokers; among them, 44.3% smoked I Quit Ordinary Smoking (IQOS), 14.8% smoked cigarettes, 10.2% smoked waterpipe (WP), 12.5% smoked cigarettes and WP, and 18.2% smoked IQOS and WP.

According to the Pittsburgh Sleep Quality Index (PSQI), 34.3% of participants had poor sleep quality. Smokers had 12.5 times higher odds of experiencing poor sleep quality compared to non-smokers (adjusted odds ratio OR_adj _= 12.58 with 95% confidence interval [CI] of 7.07-22.36; p-value <0.001). In addition, smoking a combination of two types of tobacco products (cigarettes with WP or IQOS with WP) posed the highest risk of poor sleep quality, with an adjusted odds ratio of 31.54 (95% CI of 9.15-45.74, p-value <0.001).

Elevated Fagerström Test for Nicotine Dependence (FTND) and Lebanon Waterpipe Dependence Scale (LWDS-11) scores indicated an increased risk of poor sleep quality (adjusted odds ratio OR_adj _= 4.69 with 95% CI of 2.179-10.10; p-value <0.001; and adjusted odds ratio OR_adj _=1.27 with 95% CI of 1.04-1.55; p-value 0.019, respectively).

Conclusion

Our study found a significant association between nicotine dependence and poor sleep quality among medical residents and fellows, with smokers being more susceptible to sleep disturbances. The high prevalence of IQOS smoking among medical residents and fellows in Lebanon highlights the urgent need for comprehensive research investigating the effects of heated tobacco products. Furthermore, our study reveals a critical insight into the potential additive effects of nicotine, suggesting that the concurrent use of multiple tobacco products may further elevate the risk of poor sleep quality. Recognizing the implications of our findings, it is imperative to develop targeted interventions and educational programs that promote healthier sleep habits and facilitate smoking cessation among medical residents and fellows.

## Introduction

The global prevalence of tobacco smoking and nicotine addiction has been a persistent concern, giving rise to a myriad of health-related challenges [1,2]. It is essential that future physicians be well-informed about the detrimental effects of tobacco smoking in order to help patients avoid preventable risks of tobacco. Surprisingly, studies have shown a notable prevalence of smoking among medical students, emphasizing the importance of providing them with appropriate guidance and orientation [2]. A study conducted by La Torre et al. (2012) found that the prevalence of tobacco smoking among medical students was higher than the general population with over 29.3% of medical students in Europe reporting smoking tobacco despite that more than two-thirds of them believe that health professionals are role models for patients [1].

In addition to its association with several pathologies, especially cardiovascular diseases and cancers, nicotine dependence can have negative impacts on cognitive function and job performance, with several studies linking tobacco use to decreased concentration, impaired memory, and slower reaction times [2,3]. These impairments can have serious consequences for physicians, who are responsible for making important decisions that affect the health and well-being of their patients [3].

Nicotine addiction can also disrupt the normal sleep cycle by altering the levels of neurotransmitters in the brain, such as acetylcholine, dopamine, and serotonin which are responsible for regulating sleep and wakefulness [4]. A study conducted by Almojali et al. (2017) found that approximately 76% of medical students at King Saud bin Abdulaziz University for Health Sciences in Riyadh, Saudi Arabia, reported poor sleep quality [5].

The impact of poor sleep quality and nicotine addiction on the health and cognitive function of physicians has been well documented. Vela-Bueno and his colleagues found that physicians with poor sleep quality were more likely to report lower levels of job satisfaction and higher levels of burnout [6]; similarly, a study by Kalmbach DA et al. (2017) found that medical residents who reported poor sleep quality were more likely to make errors that could have serious consequences for patients and may result in harm or even death [7].

There are various mechanisms through which smoking and nicotine dependence can impact the quality of sleep. Nicotine acts on the central nervous system (CNS) as a stimulant that interferes with the natural sleep-wake cycle of the body resulting in increased time taken to fall asleep. It can also elevate heart rate and blood pressure, making it challenging to initiate and maintain sleep [8]. Additionally, nocturnal cravings and withdrawal symptoms including anxiety and irritability associated with nicotine dependence can further contribute to sleep disruptions. Moreover, exposure to second-hand smoke has also been associated with poorer sleep quality [8,9].

A multitude of tobacco products are widely available, with cigarettes, waterpipes (WPs), and IQOS smoking being the most commonly used among the Lebanese population. WP smoking can be a significant source of nicotine, as a single session with unflavored tobacco can contain the equivalent nicotine of 70 regular cigarettes [10]. On the other hand, IQOS is a novel tobacco product that operates through heating rather than burning, marketed as a hybrid between traditional and electronic cigarettes. It is designed to significantly reduce the levels of harmful components compared to traditional cigarettes [11]. By heating tobacco to 350°C, IQOS produces a vapor with nearly 90% fewer toxic substances than cigarette smoke. However, despite its appealing technological design, there is limited research-based evidence to support its safety, especially regarding its appeal to the youth and the lack of universally accepted risk assessment. While IQOS may be less harmful than traditional cigarettes, there are still toxic chemicals present in the smoke that could potentially encourage smoking initiation. Furthermore, concerns arise about the presence of polycyclic aromatic hydrocarbons in the IQOS aerosol, suggesting possible tobacco burning and potential harm to users [11]. Another significant factor contributing to its high plausibility is that smoking IQOS is not prohibited in all enclosed areas in Lebanon, further amplified by the availability of various flavor options.

Unlike previous studies that focused solely on nicotine addiction or sleep quality among medical students, and typically included only the first three years of academic medicine, our study aims to investigate the prevalence of both poor sleep quality and nicotine addiction, their association as well as the potential moderating effects of age, gender, coffee consumption, medical year, physical activity, years of smoking, and the type of tobacco product used.

## Materials and methods

Study design and population

A cross-sectional study was conducted at the Faculty of Medicine at the Lebanese University between January and March 2023 using an online questionnaire. All residents and fellows enrolled at the Lebanese University for the academic year 2022-2023, working in different hospitals in Lebanon, and who agreed to participate in the study were included. However, the following participants were excluded from this study: being pregnant or lactating, having a known history of sleep disorders such as insomnia, parasomnia, obstructive sleep apnea, restless legs syndrome, or narcolepsy, having any type of addiction other than nicotine, and currently suffering from an ongoing illness.

Sample size calculation

The minimum sample size was calculated using an online minimum sample size generator. Assuming that the number of fellows and residents for the academic year 2022-2023 at the Faculty of Medical Sciences was estimated at 440, the required calculated sample size was 206 with a confidence level of 95%, a 5% margin of error, and 50% of expected prevalence. 

Data collection and instrumentation

Data were collected using an online survey. The questionnaire was administered via Google Forms, and a direct link was sent in both English and Arabic languages to approximately 440 participants via social media using the snowball technique. We collected a total of 360 responses, of which 10 were excluded for ineligibility.

The questionnaire was divided into four sections. The first section included baseline characteristics including age, gender, medical year, coffee consumption, physical activity, number of shifts covered, smoking status, years of smoking, and tobacco products. The smoking status included two groups: smokers (those who currently used tobacco products at least once in the past 30 days) and non-smokers [12].

The second section included the Fagerström Test for Nicotine Dependence (FTND), a widely used tool to assess the severity of nicotine addiction [13]. It contains six items that evaluate the quantity of cigarette consumption, the compulsion to use, and dependence. In scoring the FTND, yes/no items are scored from 0 to 1 and multiple-choice items are scored from 0 to 3. The items are summed to yield a total score of 0-10. This scale has a Cronbach’s alpha coefficient of 0.69 according to a study done by Browne et al. in 2018 [14]. A score of 5 or more indicates a significant dependence, while a score of 4 or less shows a low dependence [13,15].

In the third section, the Lebanon Waterpipe Dependence Scale (LWDS-11) was used as it is specifically designed to assess the level of dependence on WP smoking, especially among the Lebanese population. It includes 11 items measured on a 4-point Likert scale ranging from 0 to 3. High WP dependence was defined as having a score of LWDS-11 ≥10 [16]. In a study published in 2021, Hallit et al. estimated Cronbach's alpha coefficient to be 0.888 [17].

Finally, the last section included the Arabic Pittsburgh Sleep Quality Index (PSQI) which is a reliable and valid tool that measures sleep quality over the last one-month period. It consists of 17 self-rated questions that are subsumed within seven component scores: (1) subjective sleep quality, (2) sleep latency, (3) sleep duration, (4) habitual sleep efficiency, (5) sleep disturbances, (6) use of sleep medications, and (7) daytime dysfunction due to sleepiness. Each component was scored 0-3. A global score can be obtained from the summation of the seven components that range from 0 to 21 with higher scores denoting poorer sleep quality [18,19]. The PSQI score was categorized into two groups for analysis: good sleep quality (PSQI ≤ 5) and poor sleep quality (PSQI > 5), using a predetermined cutoff point established by Buysse et al. (1989). This cutoff point demonstrated a sensitivity of 89.6% and a specificity of 86.5% in distinguishing between individuals with good and poor sleep quality [18]. The PSQI exhibits good internal consistency with a Cronbach's alpha coefficient of 0.83 for the seven components, strong test-retest reliability, and validity as reported by Mollayeva and colleagues in 2016 [20].

Pilot study

The survey was pilot tested in a sample of five residents to check the clarity and readability of all items. They did not report any problems in understanding the questionnaire. On average, the survey was completed within approximately 10-15 minutes. The data from the pilot study were removed from the final analysis.

Statistical analysis

Statistical analysis was carried out using the Statistical Package for the Social Sciences (SPSS) version 22 (IBM Corp, Armonk, NY). Descriptive statistics were reported using means and standard deviations (SD) for continuous variables and frequency with percentages for categorical variables. Statistical bivariate analysis was performed using Pearson’s chi-square test for categorical variables and an independent student t-test for continuous variables.

Forward stepwise binominal logistic regression was used to determine the predictors of sleep quality. Adjusted odds ratio and 95% confidence intervals were reported. The logistic regression model was reached after ensuring the adequacy of our data using the Hosmer and Lemeshow test. A p-value <0.05 was considered statistically significant.

## Results

Characteristics of the study participants

The baseline characteristics of our study population are shown in Tables 1, 2. A total of 350 residents and fellows participated in the survey, and among them (43.7%) were males. Their mean age was 27.65 ± 1.71 years ranging from 25 to 31 years.

**Table 1:** Characteristics of the overall study population, non-smokers and smokers Sleep quality based on Pittsburgh Sleep Quality Index (PSQI); PGY: post-graduate year; SD: standard deviation; n: number; %: the percentage of subjects; h/w: hour per week; h/d: hour per day. *p-Value <0.05 is considered significant.

Variable	Overall (N = 350)	Non-smokers (N = 262)	Smokers (N = 88)	p-Value
Age, mean ± SD	27.65 ± 1.71	27.62 ± 1.87	27.75 ± 1.05	<0.001*
Gender, n (%)				
Male	153 (43.7%)	109 (41.6%)	44 (50%)	0.106
Female	197 (56.3%)	153 (58.4%)	44 (50%)	0.106
Post-graduate year (PGY), n (%)				
PGY 1	73 (20.9%)	67 (25.6%)	6 (6.8%)	<0.001*
PGY 2	80 (22.9%)	54 (20.6%)	26 (29.5%)	<0.001*
PGY 3	80 (22.9%)	46 (17.6%)	34 (38.6%)	<0.001*
PGY 4	67 (19.1%)	45 (17.2%)	22 (25%)	<0.001*
PGY 5	38 (10.9%)	38 (14.5%)	0 (0%)	<0.001*
PGY 6	12 (3.4%)	12 (4.6%)	0 (0%)	<0.001*
Coffee or caffeinated drinks consumption per day, n (%)				
None	61 (17.4%)	43 (16.4%)	18 (20.5%)	0.655
One cup/day	282 (80.6%)	211 (80.5%)	68 (77.3%)	0.655
2-3 cups/day	7 (2%)	8 (3.1%)	2 (2.3%)	0.655
Physical activity per day, n (%)				
Minimal (0-2 h/w)	126 (36%)	60 (23%)	66 (75%)	0.500
Moderate (3-7 h/w)	200 (57%)	185 (34%)	15 (17%)	0.500
Heavy (>1 h/d)	24 (7%)	17 (6.5%)	7 (8%)	0.500
Night shifts/month, mean ± SD	6.30 ± 0.91	6.27 ± 0.91	6.37 ± 0.91	0.777
Sleep quality based on Pittsburgh Sleep Quality Index (PSQI), n (%)				
Good (PSQI ≤5)	230 (65.7%)	209 (79.8%)	21 (23.9%)	<0.001*
Poor (PSQI >5)	120 (34.3%)	53 (20.2%)	67 (76.1%)	<0.001*

**Table 2:** Smoking characteristics LWDS-11: Lebanon Waterpipe Dependence Scale; FTND: Fagerström Test for Nicotine Dependence; WP: waterpipe; IQOS: I Quit Ordinary Smoking; SD: standard deviation; n: number; %: the percentage of subjects.

Variables	Value
Years of smoking, mean ± SD	2.73 ± 0.95
Type of tobacco products, n (%)	
Cigarettes	13 (14.8%)
WP	9 (10.2%)
IQOS	39 (44.3%)
Cigarettes + WP	11 (12.5%)
IQOS + WP	16 (18.2%)
(Cigarettes or IQOS) + (WP)	27 (30.7%)
Other types (cigars, cigarillo, pipe, vape…)	0 (0%)
FTND, mean ± SD	5.30 ± 1.60
LWDS-11, mean ± SD	14.91 ± 6.58

The majority of participants (80.6%) reported that they drank one cup of coffee per day, while 2% drank 2-3 cups per day, and 17.4% did not drink coffee at all. One quarter (25.1%) of the participants were smokers, among them 44.3% smoked I Quit Ordinary Smoking (IQOS), 14.8% smoked cigarettes, 10.2% smoked WP, 12.5% smoked cigarettes and WP, and 18.2% smoked IQOS and WP. Participants who smoked cigarettes or IQOS (either alone or in combination with other types) completed the FTND score, the overall mean FTND score was 5.30 ± 1.60. Similarly, those who smoked WP (either alone or in combination with other types) completed the LWDS score, and the overall mean LWDS score was 14.91 ± 6.58.

In our sample, the internal consistency index obtained by means of Cronbach’s alpha coefficient was 0.71 for the FTND score, 0.93 for the LWDS-11 score, and 0.92 for the PSQI score.

Table 3 provides a detailed description of the PSQI components. Based on the PSQI, 34.3% of the participants exhibited poor sleep quality, while 65.7% had good sleep quality. There is a positive correlation between smoking and all PSQI components (p-value <0.001).

**Table 3:** PSQI components Sleep quality based on Pittsburgh Sleep Quality Index (PSQI); SD: standard deviation; n: number; %: the percentage of subjects. *p-Value <0.05 is considered significant.

Variable	Overall (N = 350)	Non-smokers (N = 262)	Smokers (N = 88)	p-Value
PSQI components, mean ± SD				
Component 1: Subjective sleep quality	0.82 ± 1.19	0.35 ± 0.83	2.19 ± 1.01	<0.001*
Component 2: Sleep latency	1.44 ± 0.81	1.16 ± 0.53	2.28 ±0.89	<0.001*
Component 3: Sleep duration	1.41 ± 0.80	1.14 ± 0.52	2.18 ±0.96	<0.001*
Component 4: Sleep efficiency	0.66 ± 1.10	0.32 ± 0.77	1.63 ± 1.31	<0.001*
Component 5: Sleep disturbance	0.44 ± 0.61	0.24 ± 0.51	1.02 ± 0.45	<0.001*
Component 6: Use of sleep medication	0.41 ± 0.56	0.20 ± 0.42	1.02 ± 0.45	<0.001*
Component 7: Daytime dysfunction	0.70 ± 0.33	0.50 ± 0.29	0.14 ± 0.41	<0.001*
Total PSQI	5.25 ± 4.80	3.49 ± 3.42	10.48 ± 4.46	<0.001*
Overall sleep quality, n (%)				
Good (PSQI ≤5)	230 (65.7%)	209 (79.8%)	21 (23.9%)	<0.001*
Poor (PSQI >5)	120 (34.3%)	53 (20.2%)	67 (76.1%)	<0.001*

The association between the characteristics of the participants and sleep quality

Table 4 presents the results of the bivariate analysis, examining the association between different independent variables and sleep quality.

**Table 4:** Association between characteristics of the participants and sleep quality Sleep quality based on Pittsburgh Sleep Quality Index (PSQI); LWDS-11: Lebanon Waterpipe Dependence Scale; FTND: Fagerström Test for Nicotine Dependence; WP: waterpipe; IQOS: I Quit Ordinary Smoking; PGY: post-graduate year; SD: standard deviation; n: number; %: the percentage of subjects; h/w: hour per week; h/d: hour per day. *p-Value <0.05 is considered significant.

Variable	Poor sleep quality (N = 120)	Good sleep quality (N = 230)	p-Value
Age, mean ± SD	27.79 ± 1.28	27.58 ± 1.89	0.223
Gender, n (%)			
Male	53 (44.2%)	100 (43.5%)	0.49
Female	67 (55.8%)	130 (56.5%)	0.49
Post-graduate year (PGY), n (%)			
PGY 1	10 (8.4%)	63 (27.4%)	<0.001*
PGY 2	38 (31.9%)	42 (18.3%)	<0.001*
PGY 3	39 (32.8%)	41 (17.8%)	<0.001*
PGY 4	27 (22.7%)	40 (17.4%)	<0.001*
PGY 5	2 (1.7%)	35 (15.2%)	<0.001*
PGY 6	3 (2.5%)	9 (3.9%)	<0.001*
Physical activity per day, n (%)			
Minimal (0-2 h/w)	60 (50%)	66 (29%)	0.070
Moderate (3-7 h/w)	53 (44%)	147 (64%)	0.070
Heavy (>1 h/d)	7 (5.8%)	17 (7%)	0.070
Coffee or caffeinated drinks consumption per day, n (%)			
None	24 (20%)	37 (16.1%)	0.642
One cup/day	93 (77.5%)	186 (80.9%)	0.642
2-3 cups/day	3 (2.5%)	7 (3%)	0.642
Smoking status, n (%)			
Smoker (n = 88)	67 (55.8%)	21 (9.1%)	<0.001*
Non-smoker (n = 262)	53 (44.2%)	209 (90.9%)	<0.001*
Night shifts/month, mean ± SD	6.30 ± 0.92	6.29 ± 0.91	0.951
Years of smoking, mean ± SD	2.74 ± 0.94	2.71 ± 1.00	0.560
Type of tobacco products, n (%)			
Cigarettes	8 (6.7%)	5 (2.2%)	<0.001*
WP	6 (5%)	3 (1.3%)	<0.001*
IQOS	29 (24.2%)	10 (4.3%)	<0.001*
Cigarettes + WP	11 (9.2%)	0 (0%)	<0.001*
IQOS + WP	13 (10.8%)	3 (1.3%)	<0.001*
Non-smoker	53 (44.2%)	209 (90.9%)	<0.001*
FTND, mean ± SD	5.77 ± 1.47	3.72 ± 0.82	<0.001*
LWDS-11, mean ± SD	16.26 ± 6.11	8.16 ± 4.57	0.004*

Our results showed that post-graduate year, smoking status, and type of tobacco products are highly associated with sleep quality (p-value <0.001 for all).

Moreover, we found that participants who had exhibited a high mean FTND (5.77 ± 1.47) score and high mean LWDS score (16.26 ± 6.11) had poor sleep quality compared to those with mild nicotine dependence (p-value < 0.001 for FTND and p-value 0.004 for LWDS, respectively).

Our findings reveal no statistically significant association between sleep quality and age, gender, daily consumption of coffee or caffeinated drinks, physical activity, number of night shifts covered over the last month, and years of smoking (p-value >0.05).

Factors associated with poor sleep quality

Results from the logistic regression showed that smoking status, type of tobacco products, FTND, and LWDS scores were significantly associated with poor sleep quality. Smokers had 12.5 times higher odds of experiencing poor sleep quality compared to non-smokers (adjusted odds ratio = 12.58 with 95% CI of 7.07 to 22.36, p-value <0.001). Among smokers, the combination of two types of tobacco products (cigarettes with WP or IQOS with WP) posed the highest risk of poor sleep quality, with an adjusted odds ratio of 31.54 (95% CI of 9.15 to 45.74, p-value <0.001). Smoking only IQOS was also associated with poor sleep quality, with an adjusted odds ratio of 11.43 (95% CI of 5.24 to 24.93, p-value <0.001). Our results also showed that PGY2, PGY3, and PGY4 had poor sleep quality compared to PGY1 (adjusted odds ratio 5.7 with 95% CI of 2.56 to 12.66, p-value <0.001; adjusted odds ratio 5.99 with 95% CI of 2.69 to 13.31, p-value <0.001; adjusted odds ratio 4.25 with 95% CI of 1.86 to 9.72, p-value 0.001, respectively) (Table 5).

**Table 5:** Factors associated with poor sleep quality among the total population Variables included in the test: age, gender, post-graduate year (PGY), coffee or caffeinated drinks consumption per day, number of night shifts covered over the last month, smoking status, years of smoking, and type of tobacco products. Sleep quality based on Pittsburgh Sleep Quality Index (PSQI); WP: waterpipe; IQOS: I Quit Ordinary Smoking; PGY: post-graduate year; OR_adj_: adjusted odds ratio; 95% CI: 95% confidence interval; vs: versus. *p-Value <0.05 is considered significant.

Independent variables		Adjusted OR (OR _adj_)	95% CI	p-Value
Post-graduate year (PGY)	PGY2 vs PGY1	5.70	2.56 to 12.66	<0.001*
	PGY3 vs PGY1	5.99	2.69 to 13.31	<0.001*
	PGY4 vs PGY1	4.25	1.86 to 9.72	0.001*
	PGY5 vs PGY1	0.36	0.07 to 1.73	0.203
	PGY6 vs PGY1	2.10	0.48 to 9.10	0.322
Smoking status	Smokers vs non-smokers	12.58	7.07 to 22.36	<0.001*
Type of tobacco products	Cigarettes vs non-smokers	6.30	1.98 to 20.07	0.002*
	IQOS vs non-smokers	11.43	5.24 to 24.93	<0.001*
	WP vs non-smokers	7.88	1.91 to 32.57	0.004*
	(Cigarettes +WP or IQOS+WP ) vs non-smokers	31.54	9.15 to 45.74	<0.001*

Furthermore, we explored the association between sleep quality, nicotine dependence, and the type of tobacco smoking among smokers (Table 6).

**Table 6:** Predictors of sleep quality (PSQI total score) and sleep quality in cigarette smokers Sleep quality based on Pittsburgh Sleep Quality Index (PSQI); LWDS-11: Lebanon Waterpipe Dependence Scale; FTND: Fagerström Test for Nicotine Dependence; WP: waterpipe; IQOS: I Quit Ordinary Smoking; β: unstandardized coefficients; OR_adj_: adjusted odds ratio; 95% CI: 95% confidence interval; vs: versus. *p-Value <0.05 is considered significant.

Variable	PSQI total score		Sleep quality	
	β (95% CI)	p-Value	OR_adj_ (95% CI)	p-Value
FTND	2.46 (1.67 to 3.25)	<0.001*	4.69 (2.17 to 10.10)	<0.001*
LWDS-11	1.26 (1.39 to 2.12)	0.005*	1.27 (1.04 to 1.55)	0.019*
IQOS vs cigarettes smokers	2.28 (-0.39 to 4.96)	0.094	1.81 (0.48 to 6.84)	0.380
WP vs cigarette smokers	1.16 (0.79 to 2.46)	0.526	1.25 (0.21 to 7.41)	0.806
(Cigarettes + WP or IQOS + WP ) vs cigarette smokers	3.94 (1.12 to 6.77)	0.007*	5 (1.97 to 25.77)	0.04*

Having a high FTND and LWDS dependence score indicated an increased risk of poor sleep quality (adjusted odds ratio = 4.69 with 95% CI of 2.17 to 10.10, p-value <0.001; and adjusted odds ratio = 1.27 with 95% CI of 1.04 to 1.55, p-value 0.019, respectively). Furthermore, linear regression analyses showed that poor sleep quality was positively associated with nicotine dependence (β = 2.46, 95% CI of 1.67 to 3.25, p-value <0.001 for FTND and β = 1.26, 95% CI of 1.39 to 2.12, p-value 0.005 for LWDS-11). Our results among smokers also showed that cigarettes and WP or IQOS and WP smokers experienced poor sleep quality compared to cigarette smokers (adjusted odds ratio = 5 with 95% CI of 1.97 to 25.77, p-value 0.04).

## Discussion

To the best of our knowledge, this study represents the first examination of the prevalence of poor sleep quality and nicotine addiction, as well as their association, among Lebanese residents and fellows. Additionally, it addresses a significant knowledge gap regarding the prevalence and health implications of IQOS smoking, which remains understudied in Lebanon and the wider Arabic world, despite its popularity among young individuals. Given the absence of stringent smoking restrictions and tobacco advertising regulations in Lebanon, it becomes imperative to conduct research starting with future physicians who play a crucial role in guiding and educating the general population about the multifaceted risks associated with tobacco smoking [17,21].

The smoking prevalence among our study population was approximately 25%, which aligns with the prevalence reported by Chidiac et al. in their 2016 study among Lebanese medical students [21]. Furthermore, our study reveals a prevalence of poor sleep quality of 34.3%, with smokers being 12.58 times more likely to experience poor sleep quality compared to non-smokers. Similarly, Abdulghani et al. (2012) found that 36.6% of medical students exhibited abnormal sleep habits; however, their study was limited to students in the first, second, and third academic years [22]. Additionally, Liao et al. in their study in 2012 in rural and urban areas of Hunan province, China, reported that smokers demonstrated poorer sleep quality compared to non-smokers, as measured by the PSQI but this study was confined only to cigarette smoking without assessing the nicotine dependence among smokers [23].

Interestingly, we have found that individuals who used a combination of two tobacco products exhibited higher odds of having poor sleep quality in comparison to those using a single type of product, indicating a possible additive effect of nicotine on sleep quality, particularly among individuals using multiple tobacco products. Notably, no previous studies have investigated the specific impact of smoking one versus two types of tobacco products, underscoring the novelty and significance of this study in shedding light on this relationship.

Regarding the type of tobacco product used, it is surprising to find that IQOS smoking was more prevalent than cigarettes and WP. IQOS represents a relatively new and increasingly popular tobacco product in Lebanon, yet no prior studies have examined its prevalence in the country [11].

These results emphasize the need for interventions targeting smoking cessation among medical residents. By quitting smoking, residents can improve their overall well-being, sleep quality, and job performance within the hospital setting, thereby reducing the risks of errors and mismanagement caused by sleep deprivation [22].

Our study has some limitations. Due to the snowball sampling strategy, the findings did not represent all the Lebanese residents and fellows and therefore we cannot generalize our results, and it is merely impractical to extrapolate these findings to other Lebanese residents and fellows with different demographic characteristics. However, we intended to choose students from the Lebanese University, which is the only public university in Lebanon that encompasses students from all regions of Lebanon, and it has experienced a significant increase in enrollment over the years [24]. In fact, in certain years, students from the Lebanese University comprised 60% of the total student population in Lebanon and accounted for 38.7% of the total university graduates [24].

Moreover, the reliance on self-reported questionnaire subjected the study to information bias. Although a validated instrument was used for assessing sleep quality, objective methods of measuring sleep quality such as actigraphy and polysomnography may strengthen the results.

Finally, the cross-sectional nature of the study can only demonstrate association and not a cause-effect relationship. Therefore, it is not possible to ascertain the causality or temporal relationship concerning the pathways of association between quality of sleep and nicotine dependence which is likely to be bidirectional. Despite the limitations identified, we believe that the study addresses a major health problem that challenges physicians in Lebanon. 

## Conclusions

In conclusion, this study highlights a significant association between nicotine dependence and poor sleep quality among medical residents and fellows, indicating that smokers are more prone to experience compromised sleep compared to non-smokers. The notable prevalence of IQOS smoking among medical residents emphasizes the necessity for additional research on the effects and safety of alternative tobacco products. Moreover, this study suggests that using multiple types of tobacco products may increase the likelihood of poor sleep quality, suggesting a potential additive effect of nicotine on sleep disturbances that warrants further investigation in future studies.
